# Prognostic significance of pretreatment albumin–bilirubin (ALBI) grade and platelet–albumin–bilirubin (PALBI) grade in patients with small cell lung cancer

**DOI:** 10.1038/s41598-024-51375-2

**Published:** 2024-01-16

**Authors:** Engin Kut, Serkan Menekse

**Affiliations:** Medical Oncology Clinic of Manisa State Hospital, 45040 Manisa, Turkey

**Keywords:** Cancer therapy, Tumour biomarkers, Cancer, Lung cancer, Small-cell lung cancer, Oncology, Medical research, Biomarkers, Predictive markers, Prognostic markers, Prognostic markers, Lung cancer, Small-cell lung cancer

## Abstract

Small cell lung cancer (SCLC) is a common cancer among the world’s lung cancers. Despite advances in diagnosis and treatment, the prognosis is still poor. There is no effective biomarker other than stage in daily practice. However, in daily practice, patients may have different features and survival times even though they have the same stage. Previously, albumin–bilirubin (ALBI) grade, platelet–albumin–bilirubin (PALBI) grade were used to determine the prognosis of acute-chronic liver failure and acute upper gastrointestinal bleeding in liver cirrhosis. In subsequent studies, they were found to be associated with prognosis in hepatocellular carcinoma (HCC) and other solid cancers. However, the prognostic relationship between ALBI grade, PALBI grade, and SCLC is unknown. Therefore, we conducted this study to examine the relationship between ALBI grade and PALBI grade and prognosis in SCLC patients. Data of 138 patients with advanced SCLC at diagnosis between 2009 and 2020 were analyzed retrospectively. The results of the multivariate analysis were as follows: ALBI grade 1 vs 2, hazard ratio (HR) = 1.608, p = 0.002 for OS and HR = 1.575, p = 0.002 for PFS; ALBI grade 1 vs 3, HR = 2.035, p < 0.001 for OS and HR = 2.675, p < 0.001 for PFS; PALBI grade 1 vs 2, HR = 1.302, p = 0.006 for OS and HR = 1.674, p = 0.002 for PFS; and PALBI grade 1 vs 3, HR = 1.725, p < 0.001 for OS and HR = 2.675, p < 0.001 for PFS. In conclusion, the ALBI and PALBI grades were determined to be associated with the prognosis of SCLC, and they can be used as easy, inexpensive, and practical markers in determining the follow-up treatment and prognosis of SCLC patients.

## Introduction

Lung cancer is one of the most common cancers worldwide and one of the leading causes of cancer-related deaths^1^. Small cell lung cancer (SCLC) accounts for 15–20% of all lung cancers^2^. However, despite advances in diagnosis and treatment, patients with SCLC still have a poor prognosis and short overall survival, due to rapid relapse, short doubling times, and early distant metastasis capacity^3^. The main component of treatment is systemic chemotherapy. While chemoradiotherapy is the primary treatment in limited-stage, palliative chemotherapy is essential in extensive-stage^2^. Although poor performance score, stage of the disease, lactate dehydrogenase (LDH), weight loss, female sex, age, number of metastases, and creatinine level are associated with prognosis in studies, there is no effective biomarker other than a stage for follow-up treatment and prognosis in daily practice^3^. However, patients may have different features and survival times even if they have the same stage. Therefore, other markers that can be more standardized are needed. ALBI grade and PALBI grade are obtained based on the measurement of serum albumin and bilirubin levels and platelet counts. These grades were previously used to determine the prognosis of acute-chronic liver failure and acute upper gastrointestinal bleeding in liver cirrhosis. In subsequent studies, they were found to be associated with prognosis in hepatocellular carcinoma (HCC) and other solid cancers (pancreas, colon, and stomach cancer)^4–8^. However, to the best of our knowledge, there is no study in the literature showing the benefit of ALBI grade in predicting the prognosis of SCLC. Therefore, we conducted this study to examine the relationship between ALBI and PALBI with prognosis in patients with SCLC, who was in the extensive stage of the disease at the time of diagnosis and received cisplatin etoposide as first-line therapy.

## Methods

### Study population

The medical data of 138 patients with SCLC, who were treated and followed up at Manisa State Hospital between 2009 and 2022 were retrospectively examined. Patients aged ≥ 18 years, who had an SCLC histology, extensive stage at the time of diagnosis, received cisplatin etoposide as first-line treatment (only chemotherapy) and had complete data were included in the study. Patients with a non-SCLC histology result, active infection, obstructive jaundice, chronic liver disease, liver cirrhosis, esophageal varices, and multiple primary tumors, who received immunotherapy or retargeted therapy as primary and subsequent therapy, and those with missing data were excluded from the study**.** The stages of the patients were determined radiologically using positron emission computed tomography and brain magnetic resonance imaging. Significant progression of non-target lesions, an increase of at least 20% in the sum of the diameters of target lesions, or the emergence of one or more new lesions were considered as progression according to the response evaluation criteria in solid tumors.

### Data collection

The patient’s age, sex, Eastern Cooperative Oncology Group (ECOG) performance status score, smoking status, date of diagnosis, location of metastasis (liver, lung, distant lymph node, adrenal, brain, and bone), LDH (U/L), uric acid (UA) (mg/dL), aspartate aminotransferase (U/L), alanine aminotransferase (U/L), neutrophil (/μL), lymphocyte (/μL), platelet (/μL), albumin (g/dL), and total bilirubin (mg/dL) values, ALBI, PALBI, albumin-to-globulin ratio (AGR), and prognostic nutritional index (PNI) were obtained from the medical archive files, and the relationship of these factors with survival was examined. To obtain the overall survival (OS) times of the patients, the time from the date of diagnosis to mortality or the last follow-up for the surviving patients was calculated. Progression-free survival (PFS) was determined as the time from the beginning of treatment to disease progression or mortality from any cause.

AGR was calculated as albumin/(total proteins − albumin), ALBI score as (log10 bilirubin (μmol/L) × 0.66) + albumin (g/L) × − 0.0852, PALBI score as 2.02 × log10 total bilirubin (μmol/L) − 0.37 × (log10Total bilirubin) 2 − 0.04 × albumin (g/L) was calculated from the formula − 3.48 × log10Platelet (10^9^/L) + 1.01 × (log10Platelet) with blood values at the time of diagnosis. The median PNI score of the patients was 41.52, and their median AGR score was 1.12. The patients were divided into two groups each according to the ECOG performance score (< 2 and ≥ 2), AGR (≤ 1.12 and > 1.12), and PNI (≤ 41.52 and > 41.52), and three groups each according to the ALBI score (grade 1: ≤ − 2.60, grade 2: − 1.39 to − 2.60, and grade 3: > − 1.39) and PALBI score (grade 1: ≤ − 2.53, grade 2: − 2.09 to − 2.53, and grade 3: > − 2.09).

### Statistical analysis

Descriptive statistics were presented as mean, standard deviation, median, minimum, and maximum values for numerical variables, and as numbers and percentages for categorical variables. The comparison of numerical variables between two independent groups was performed using Student’s *t*-test in the case of a normal distribution and the Mann–Whitney *U* test otherwise. The comparison of numerical variables between more than two independent groups was made using one-way analysis of variance (ANOVA) in the case of normal distribution and the Kruskal–Wallis test otherwise. Rates were compared between the groups using the chi-square analysis and Fisher’s exact test. Survival analyses were undertaken with the Kaplan–Meier method. Determinative factors were examined using the Cox regression analysis (with backward selection). p < 0.05 was considered significant in all statistical analyses.

### Ethics approval

The study was conducted by the principles of the Declaration of Helsinki and reviewed and approved by the Health Sciences Ethics Committee of Manisa Celal Bayar University (decision number: 20.478.486/1569, date: 02.11.2022). All authors confirm that all methods were carried out by the relevant guidelines and regulations. Written informed consent was obtained from each patient.

## Results

A total of 138 patients, 98 (71%) male and 49 (29%) female, were retrospectively evaluated. The mean age of the patients was 58.94 ± 8.30 years. Of all the patients, 97 (71%) were male and 40 (29%) were female. Additionally, 54 (39.1%) patients had an ECOG score (≥ 2) of 2 or more, and 84 had an ECOG score (< 2) of less than 2 (Table 1). We calculated ALBI and PALBI grades based on the blood values of all patients at the time of diagnosis. Information about these values is shown in Table 1. All the patients were Smokers. The patients had a 40 (20–120) pack-years smoking history and all were active smokers at the time of diagnosis. Every patients received cisplatin etoposide as first-line therapy. In addition, 48 (34.78%) patients received second-line chemotherapy (irinotecan–carboplatin), and 20 (14.49%) patients received third-line chemotherapy (paclitaxel). Our median follow-up period was 13 (3–25) months. The median OS time of the patients was 11 [95% confidence interval (CI) 8.52–13.49] months, and their median PFS time was 4.48 (95% CI 2.68–6.39) months. In our study, 41 (29.71%) of our patients received less than 4 cycles of chemotherapy, and 97 (70.29%) patients received 4 or more cycles of chemotherapy. There was no statistically significant difference between the two groups. At the time of the study, 2 patients were continuing their treatment with first-line chemotherapy, 5 patients with second-line chemotherapy and 2 patients with third-line chemotherapy.

**Table 1:** Demographic, clinical, and laboratory characteristics of the patients.

	All patients	ALBI grade 1	ALBI grade 2	ALBI grade3	P value	PALBI grade 1	PALBI grade 2	PALBI grade 3	P value
Age	58.94 ± 8.30	57.96 ± 9.06	59.4 ± 7.51	62.4 ± 7.52	0.17	59.3 ± 10.88	58.98 ± 7.8	58.62 ± 8.04	0.97
Age									
≤ 65 years	39 (28.3%)	17 (43.6%)	18 (46.2)	4 (10.3%)	0.98	6 (15.4%)	22 (56.4%)	11 (28.2%)	0.84
> 65 years	99 (71.7%)	42 (43.3%)	46 (47.4%)	9 (9.3%)		14 (14.1%)	61 (61.2%)	24 (24.2%)	
Sex									
Male	98 (71%)	44 (45.4%)	43 (44.3%)	10 (10.3%)	0.59	16 (16.3%)	55 (56.1%)	27 (27.6%)	0.31
Female	40 (29%)	15 (38.5%)	21 (53.8%)	3 (7.7%)		4 (10%)	28 (70%)	8 (20%)	
ECOG performance score									
≥ 2	54 (39.1%)	20 (37%)	28 (51.9%)	6 (11.1%)	0.42	11 (20.4%)	24 (44.4%)	19 (35.2%)	0.011
< 2	84 (60.9%)	39 (47.6%)	36 (43.9%)	7 (8.5%)	0.42	9 (10.7%)	59 (70.2%)	16 (19%)	
Metastasis site									
Liver	60 (43.5%)	15 (25.4%)	33 (55.9%)	12 (18.6%)	0.01	7 (11.7%)	35 (58.3%)	18 (30%)	0.46
Bone	73 (52.9%)	30 (41.1%)	37 (50.7%)	6 (8.2%)	0.62	9 (12.3%)	43 (58.9%)	21 (28.4%)	0.54
Adrenal	40 (29%)	16 (41%)	19 (48.7%)	5 (10.3%)	0.94	4 (10%)	23 (57.5%)	13 (32.5%)	0.37
Brain	50 (36.2%)	25 (52.1%)	20 (39.6%)	5 (8.3%)	0.32	7 (14%)	28 (56%)	15 (30%)	0.64
Lung	68 (49.2%)	28 (41.4%)	36 (52.9%)	4 (5.9%)	0.21	8 (11.8%)	43 (63.2%)	18 (25%)	0.64
LN	64 (46.4%)	29 (44.4%)	29 (46%)	6 (9.5%)	0.97	10 (15.8%)	37 (57.8%)	17 (26.6%)	0.86
Laboratory values									
AST (U/L)	30 (12–115)	31.5 (18–60)	34 (12–85)	28.5 (16–125)	0.37	34.5 (20–58)	33.5 (12–115)	32 (14–98)	0.73
ALT (U/L)	33 (12–115)	34 (20–58)	33.5 (12–115)	32 (14–98)	0.51	31.5 (18–66)	34 (12–85)	28.5 (16–115)	0.34
LDH (U/L)	345 (147–978)	289 (147–835)	415 (166–978)	452 (233–598)	0.001	395 (158–658)	364 (158–978)	344 (147–845)	0.73
UA (mg/dL)	4.8 (2.5–8.7)	5 (3.2–8.7)	4.75 (2.5–8.7)	4.4 (2.9–7.7)	0.25	5.5 (2.9–7.7)	4.8 (2.5–8.7)	4.9 (2.5–7.4)	0.42
Albumin (g/dL)	3.5 (1.8–4.6)	4.4 (2.5–4.6)	3.4 (2.5–3.9)	2.4 (1.8–2.7)	0.001				
Total bilirubin (mg/dL)	0.5 (0.2–1.9)	0.4 (0.2–0.9)	0.8 (0.2–1.7)	1.2 (0.7–1.9)	0.001	0.65 (0.3–1.4)	0.5 (0.2–2.1)	0.7 (0.2–1.9)	0.62
Neutrophil (10^3^/μL)	6.45 (3.33–14.76)	6.45 (3.8–14.1)	6.31 (3.3–14.7)	6.3 (4.8–8.43)	0.97	7.6 (4.2–13.5)	6.2 (3.8–11.5)	6.5 (3.33–14.76)	0.44
Lymphocyte (10^3^/μL)	1.45 (0.9–2.3)	1.4 (0.9–2.3)	1.5 (0.9–2.2)	1.6 (0.9–2.1)	0.46	1.32 (0.9–2.1)	1.41 (0.9–2.3)	1.54 (0.9–2.16)	0.70
Platelet (10^3^/μL)	278 (132–651)	279 (132–651)	285 (216–651)	298 (104–253)	0.43	276 (216–447)	285 (119–651)	291 (134–589)	0.49
Chemotherapy cycle number									
< 4	41 (29.71%)	17 (41.46%)	20 (48.78%)	4 (9.76%)	0.810	5 (12.19%)	26 (63.41%)	10 (24.39%)	0.70
4 ≤	97 (70.29%)	45 (46.39%)	44 (45.30%)	8 (8.25%)		15 (15.46%)	54 (55.67%)	28 (28.87%)	
PNI									
> 41.52	69 (50%)	29 (43.3%)	33 (49.3%)	5 (7.4%)	0.69	10 (14.5%)	38 (55.1%)	21 (30.4%)	0.37
≤ 41.52	69 (50%)	30 (43.5%)	31 (44.9%)	8 (11.6%)		10 (14.5%)	45 (65.2%)	14 (20.3%)	
AGR									
1.12 ≥	67 (48.6%)	7 (10.8%)	45 (69.2%)	13 (20%)	0.001	10 (14.9%)	39 (58.2%)	18 (26.9%)	0.88
1.12 <	71 (51.4%)	52 (71.2%)	19 (26.8%)	0 (0%)		10 (14.1%)	44 (62%)	17 (23.9%)	

*SD* standard deviation, *ECOG* Eastern Cooperative Oncology Group, *AST* aspartate aminotransferase, *ALT* alanine aminotransferase, *LDH* lactate dehydrogenase, *UA* uric acid, *AGR* albumin-to-globulin ratio, *PNI* prognostic nutritional index, *ALBI* albumin-bilirubin grade, *PALBI* platelet–albumin–bilirubin grade.

**Figure 1:**
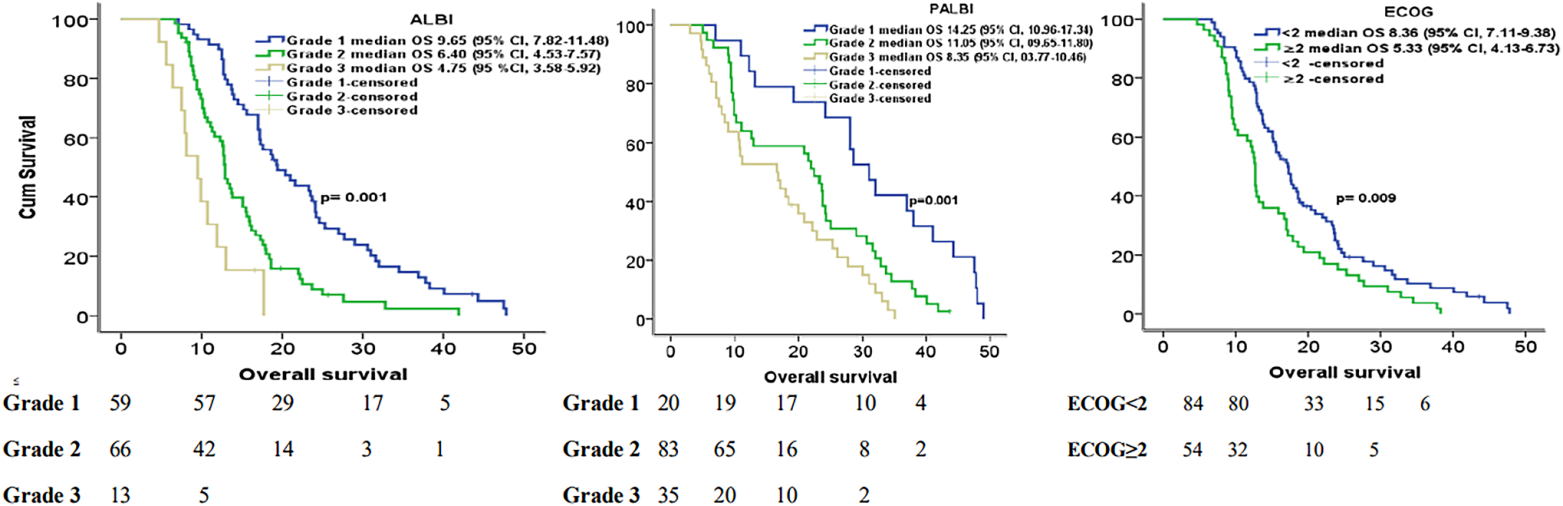
Kaplan–Meier curves of albumin–bilirubin (ALBI) grade, albumin–bilirubin and platelet (PALBI) grade and Eastern Cooperative Oncology Group (ECOG) performance score for overall survival.

**Figure 2:**
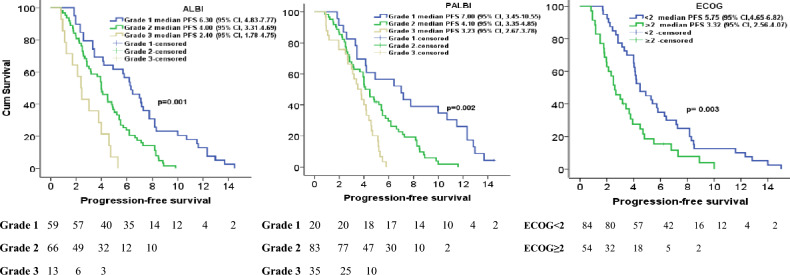
Kaplan–Meier curves of albumin–bilirubin (ALBI) grade, albumin–bilirubin and platelet (PALBI) grade and Eastern Cooperative Oncology Group (ECOG) performance score for progression-free survival.

The results of the multivariate analysis were as follows: ALBI grade 1 vs 2, hazard ratio (HR) = 1.608, p = 0.002 for OS and HR = 1.575, p = 0.002 for PFS; ALBI grade 1 vs 3, HR = 2.035, p < 0.001 for OS and HR = 2.675, p < 0.001 for PFS; PALBI grade 1 vs 2, HR = 1.302, p = 0.006 for OS and HR = 1.674, p = 0.002 for PFS; and PALBI grade 1 vs 3, HR = 1.725, p < 0.001 for OS and HR = 2.675, p < 0.001 for PFS (Tables 2 and 3) (Fig. 1 and 2).

**Table 2:** Univariate and multivariate analyses of overall survival.

	Univariate analysis (HR, 95% CI)	p value	Multivariate analysis (HR, 95% CI)	p value
Age (< 65 vs ≥ 65 years)	1.016 (0.996–1.037)	0.114		
Sex	1.061 (0.731–1.540)	0.76		
ECOG performance score (< 2 vs 2 ≤)	1.509 (1.122–2.253)	0.009	1.385 (1.271–3.178)	0.006
Liver metastasis	1.302 (0.920–1.836)	0.128		
Bone metastasis	1.046 (0.750–1.469)	0.790		
Adrenal metastasis	0.933 (0.647–1.357)	0.729		
Brain metastasis	0.911 (0.641–1.295)	0.603		
Lung metastasis	1.660 (0.830–1.635)	0.371		
LN metastasis	1.189 (0.849–1.665)	0.313		
C. Cycle number	1.165 (0.912–1.589)	0.058		
ALT (U/L)	0.999 (0.999–1.008)	0.886		
AST (U/L)	1.001 (0.991–1010)	0.882		
LDH (U/L)	0.463 (0.217–1.21)	0.092		
Uric acid (mg/dL)	0.843 (0.693–1.567)	0.843		
Albumin (g/dL)	0.513 (0.400–0.670)	0.027	1.606 (0.569–4.533)	0.371
Total bilirubin (mg/dL)	1.67 (0.094–2.540)	0.042	0.330 (0.099–1.098)	0.71
Neutrophil (U/L)	1.000 (1.000–1.0000)	0.549		
Lymphocyte (U/L)	1.000 (1.000–1.000)	0.657		
Platelet (U/L)	1.000 (0.998–1.001)	0.543		
AGR (≤ 1.12 vs > 1.12)	1.47 (1.049–2.078)	0.01	1.978 (0.865–2.275)	0.12
PNI (≤ 41.52 vs > 41.52)	1.304 (1.02–1.59)	0.025	1.098 (0.925–1.652)	0.65
ALBI		= 0.001		
Grade 1 vs 2	2.030 (1.639–3.6100)	< 0.001	1.608 (1.320–3.932)	= 0.002
Grade1 vs 3	3.680 (2.600–9.733)	< 0.001	2.035 (1.465–5.710)	< 0.001
PALBI		= 0.001		
Grade 1 vs 2	2.029 (1.409–2.926)	< 0.001	1.302 (1.160–3.093)	= 0.006
Grade1 vs 3	3.723 (1.832–6.740)	< 0.001	1.725 (1.281–4.910)	< 0.001

*HR* hazard ratio, *CI* confidence interval, *ECOG* Eastern Cooperative Oncology Group, *LN* lymph node, *C.* *Cycle number* chemotherapy cycle number, *AGR* albumin–globulin ratio, *PNI* prognostic nutritional index, *ALBİ* albumin–bilirubin grade, *AST* aspartate aminotransferase, *ALT* alanine aminotransferase, *LDH* lactate dehydrogenase.

**Table 3:** Univariate and multivariate analyses of progression-free survival.

	Univariate analysis (HR, 95% CI)	p value	Multivariate analysis (HR, 95% CI)	p value
Age (< 65 vs ≥ 65 years)	1.308 (0.907–1.900)	0.159		
Sex	1.229 (0.849–1.781)	0.275		
ECOG performance score (< 2 vs 2 ≤)	1.720 (1.478–2.000)	0.003	1.385 (1.271–3.178)	0.006
Liver metastasis	1.194 (0.844–1.691)	0.317		
Bone metastasis	1.144 (0.817–1.602)	0.433		
Adrenal metastasis	1.137 (0.785–1.646)	0.496		
Brain metastasis	1.179 (0.830–1.676)	0.358		
Lung metastasis	1.279 (0.090–1.789)	0.580		
LN metastasis	1.400 (0.811–1,601)	0.451		
C. Cycle number	1.282 (0.943–1.746)	0.054		
ALT (U/L)	1.007 (0.999–1.016)	0.086		
AST (U/L)	1.007 (0.895–1.021)	0.109		
LDH (U/L)	1.000 (0.999–1.006)	0.585		
Uric acid (mg/dL)	1.059 (0.935–1.200)	0.367		
Albumin (g/dL)	0.594 (0.462–0.762)	0.016	0.935 (0.888–0.985)	0.012
Total bilirubin (mg/dL)	1.124 (1.012–1.323)	0.042		
Neutrophil (10^3^/μL)	1.000 (0.999–1.007)	0.085		
Lymphocyte (10^3^/μL)	0.999 (0.999–1.00)	0.020	0.999 (0.998–1.000)	0.042
Platelet (10^3^/μL)	1.001 (1.000–1.003)	0.129		
AGR (1.12 ≥ vs 1.12 <)	1.255 (0.618–2.540)	0.53		
PNI (41.52 ≥ vs 41.52 <)	1.194 (1.020–1.368)	0.047	1.004 (0.973–1.037)	0.29
ALBI		= 0.001		< 0.001
Grade 1 vs 2	2.226 (1.392–3.5610)	< 0.001	1.575 (1.230–4.273)	= 0.002
Grade1 vs 3	3.512 (1.912–6.210)	< 0.001	2.675 (1.865–6.710)	< 0.001
PALBI		= 0.002		< 0.001
Grade 1 vs 2	2.144 (1.344–3.410)	< 0.001	1.674 (1.340–4.693)	= 0.002
Grade1 vs 3	3.420 (1.820–5.230)	< 0.001	2.675 (1.781–7.120)	< 0.001

*HR* hazard ratio, *CI* confidence interval, *ECOG* Eastern Cooperative Oncology Group, *LN* lymph node, *C. Cycle number* chemotherapy cycle number, *AGR* albumin–globulin ratio, *PNI* prognostic nutritional index, *ALBİ* albumin–bilirubin grade, *AST* aspartate aminotransferase, *ALT* alanine aminotransferase, *LDH* lactate dehydrogenase.

## Discussion

In recent years, significant progress has been made in the treatment of non-small cell lung cancer with the increase in molecular biology techniques, targeted therapies, and immunotherapy. However, SCLC still has a poor prognosis The average survival time is two to four months in untreated patients and can extend to 10 months in extensive disease stage and 24–36 months in limited stage with the use of platinum/etoposide or platinum/irinotecan regimens^2,9^.

Recent studies have shown that malnutrition, chronic inflammation, and chronic inflammation secondary to the secretion of proinflammatory cytokines begin catabolic cytokine production processes, such as the malignant transformation of cells in the suppression of the immune response and neoangiogenesis secondary to the inflammatory status. All of these may indicate a complex relationship between immunity, inflammation and nutritional status^10–12^.

Albumin is a protein synthesized from the liver and a negative acute phase reactant. It is affected by the presence of inflammation. In malnutrition and cachexia, the blood level may change depending on the released cytokines. Therefore, albumin can reflect inflammatory and immune states and nutritional status^13^.

Bilirubin is a product of heme metabolism and play a major role in the antioxidant mechanism. It is also known that bilirubin is involved in intestinal homeostasis and host defense. Therefore, it also plays a significant role in shaping the intestinal microbiota. Changes in the intestinal microbiota may play an important role in the cancer microenvironment, especially in the development of colon cancer. It has been found to play a role in carcinogenesis as well as in the prognosis of some cancers^8,14^.

Platelets play a role in hemostasis and thrombosis, as well as in inflammation by secreting proinflammatory cytokines, such as platelet-derived growth factor (PDGF) and vascular endothelial growth factor (VEGF). In addition, they are involved in the formation of the tumor microenvironment by causing the migration of inflammatory cells to the inflammation area, escape of tumor cells from the immune system due to the tumor microenvironment, angiogenesis required for cancer development and metastasis, and progression of cancer^15^. Therefore, there is a complex relationship between the inflammatory, immune, and nutritional status of patients and the later steps of carcinogenesis.

ALBI and PALBI grades can indicate cancer prognosis since they consist of albumin and bilirubin levels and platelet count. ALBI and PALBI were first found to be associated with prognosis in cirrhosis and varicose bleeding due to chronic liver disease. In later studies, these grades were also associated with the prognosis of HCC. Subsequently, these grades were found to be related to prognosis in pancreatic cancer with liver metastasis, operated gastric cancer, cholangiocellular carcinoma, and non-small cell lung cancer^16–19^.

Although previous studies have established a relationship between ALBI and PALBI grades and many cancers^16–19^, the relationship between these grades and prognosis in patients with SCLC has not yet been examined. In our study, the median OS and PFS were linked to ALBI and PALBI grades. Our study is the first in the literature to demonstrate a relationship between ALBI and PALBI grades and SCLC prognosis.

Immunotherapy + chemotherapy is the standard first line treatment for small cell lung cancer. Our study would have been more useful if it had been conducted in patients receiving immunotherapy + chemotherapy, which is standard today. In this case, although the data obtained seems to be theoretically useful in a limited number of patients, the number of patients who cannot receive immunotherapy + chemotherapy is not small. These grades can be used respectively according to the following situations in daily practice, many patients in our country and in many other parts of the world cannot access immunotherapies due to socio-economic reasons. For this reason, the first-line treatment of patients is still mostly chemotherapy. Therefore, it can be used to determine the prognosis in this group of patients. In addition, although immunotherapy is recommended as standard for patients with small cell lung cancer, chemotherapy is also recommended after it progresses. There are a considerable number of patients in this group. Although our study is a study showing the relationship between ALBI, PALBI grade and prognosis in patients receiving first-line chemotherapy, its results may also be prognostic in patients who will receive chemotherapy in case of progression after the first line. Additionally, immunotherapies are contraindicated in some diseases. For this reason, it cannot be used in this group of patients. In this case (in some chronic diseases where immunotherapies are contraindicated, Multiple sclerosis, Inflammatory bowel disease, etc.), Chemotherapy is used. Since these chronic diseases are very common, it may be useful to obtain information about the prognosis in this group where chemotherapy is recommended.

Although our study is limited by the retrospective single-center design and small number of patients, it is important because it is the first in the literature to show the relationship between ALBI and PALBI grades and prognosis in patients with SCLC.

In conclusion, ALBI and PALBI grades were determined to be associated with the prognosis of SCLC. These grades can be used as easy, inexpensive, and practical markers in determining the follow-up treatment and prognosis of patients with SCLC.

## Data Availability

The datasets generated during and/or analyzed during the current study are available from the corresponding author upon reasonable request.
